# Cyanogenic Pseudomonads Influence Multitrophic Interactions in the Rhizosphere

**DOI:** 10.1371/journal.pone.0002073

**Published:** 2008-04-30

**Authors:** Thimmaraju Rudrappa, Robert E. Splaine, Meredith L. Biedrzycki, Harsh P. Bais

**Affiliations:** 1 Department of Plant and Soil Sciences, University of Delaware, Newark, Delaware, United States of America; 2 Delaware Biotechnology Institute, Newark, Delaware, United States of America; Cairo University, Egypt

## Abstract

In the rhizosphere, plant roots cope with both pathogenic and beneficial bacterial interactions. The exometabolite production in certain bacterial species may regulate root growth and other root-microbe interactions in the rhizosphere. Here, we elucidated the role of cyanide production in pseudomonad virulence affecting plant root growth and other rhizospheric processes. Exposure of *Arabidopsis thaliana* Col-0 seedlings to both direct (with KCN) and indirect forms of cyanide from different pseudomonad strains caused significant inhibition of primary root growth. Further, we report that this growth inhibition was caused by the suppression of an auxin responsive gene, specifically at the root tip region by pseudomonad cyanogenesis. Additionally, pseudomonad cyanogenesis also affected other beneficial rhizospheric processes such as *Bacillus subtilis* colonization by biofilm formation on *A. thaliana* Col-0 roots. The effect of cyanogenesis on *B. subtilis* biofilm formation was further established by the down regulation of important *B. subtilis* biofilm operons *epsA* and *yqxM*. Our results show, the functional significance of pseudomonad cyanogenesis in regulating multitrophic rhizospheric interactions.

## Introduction

Cyanogenesis, a process by which cyanide (CN^−^ or HCN) is produced, has been demonstrated to occur in both bacteria and plants. Among bacteria, it has been studied extensively in fluorescent pseudomonads, especially *Pseudomonas fluorescens* and *Pseudomonas aeruginosa* [1]. In addition to its activity in *Pseudomonas*, cyanogenesis has also been reported in *Chromobacterium violaceum* and has often been reported to occur in the case of cyanobacteria such as *Anacystis nidulans*, *Nostoc muscorum* and *Plectonema boryanum* [2]–[5]. Additionally, some strains of *Rhizobium leguminosarum* have also been reported to produce cyanide as free-living bacteria [6]. In most of these cases, cyanide has been reported to be synthesized from the amino acid glycine [7]. The sorption and mobility of cyanide produced from such organisms is mainly through soil surfaces and solutions. It is known that cyanide produced in the soil is usually associated with various metal ions, allowing for its rapid mobility in the ground water and subsequent diffusion in the atmosphere [8]. The two most extensively studied bacteria for cyanogenesis, as stated earlier *P. aeruginosa* and *P. fluorescens*, are both commonly found in the soil.

*Pseudomonas aeruginosa*, a Gram-negative bacterium, has been reported to cause opportunistic infections in humans, animals and plants [9]–[13]. The ability of *P. aeruginosa* to infect humans and plants has been ascribed to its production of various secreted and surface associated virulence factors [11]. Apart from various protein toxins, *P. aeruginosa* also produces small molecular toxins such as cyanide that facilitate the overall virulence of this opportunistic bacterium against multiple hosts [14]–[21]. Interestingly, certain pseudomonads have been characterized as root colonizers of several food crops that evade pathogenesis against multiple pathogens [22]. Siderophores and cyanide production ability in various pseudomonads are linked to antagonistic and disease suppressing activity against various plant pathogens [23]. The discovery that *P. aeruginosa* has the ability to infect plants allowed for the use of the *A. thaliana - P. aeruginosa* infection model by various research groups [11], [20]. The two contrasting ecological roles of pseudomonads as a biocontrol agent and opportunist plant pathogen suggests that both root colonization and pathogenesis inflicted by this bacterium are highly species specific interactions.

Apart from bacteria, plants have also been reported to produce cyanide to defend themselves against herbivores [24]. In plants, cyanide is produced as cyanogenic glycosides and stored in the vacuole of the cell. Storage in the vacuole allows for separation from the enzymes, which act on the glycosides, and prevents the cells from auto-toxicity [24]. When cells are damaged by herbivores, the cyanogenic glycosides and the enzymes are released from their separate compartments and react to produce HCN, which is toxic to herbivores [24]. The potential of cyanogenic glycosides in plants as chemical defense has been demonstrated in *Sorghum bicolor* [25]. Additionally, cyanogenesis plays an important role in the fitness of *Trifolium repens* against herbivores [26]. The quantitative analysis of the amount of cyanide containing compounds stored in a given tissue and their capacity to release HCN per unit time as a reaction to herbivory has been studied in lima bean (*Phaseolus lunatus* L.) [27]. Despite these foliar examples, there are no reports of secretion of cyanide into the rhizosphere through plant roots. However, *P. fluorescens*, when associated with weed seedlings, are known to produce toxic levels of cyanide, causing considerable inhibition of root growth [28]–[30]. Although the potential of cyanogenesis by different strains of rhizobacteria such as *P. fluorescens* and *P. aeruginosa*, for suppression of weed growth or allelopathy has been examined, few of these studies have attempted to investigate the rhizotoxic effect of bacterial cyanogenesis [30]–[31].

Auxin is the key component which stimulates cell extension and growth in plant roots [32]. Although, the intervention of secondary metabolites such as flavonoids with auxin activity is well investigated [33], the effect of cyanide on auxin perception and biosynthesis still awaits elucidation. The effects of cyanide on the processes of auxin perception or biosynthesis cascade at the root tips are worth considering owing to its role in gibberellic acid mediated root growth and elongation [34]. Auxin plays a key role in the regulation of nuclear export and distribution of DELLA protein RGA which suppresses root growth [34]. To our knowledge, no study has attempted to elucidate the mechanism of inhibition of plant root growth by cyanogenesis in the opportunistic pathogen *P. aeruginosa* and its effect on the colonization of beneficial plant growth promoting rhizobacteria (PGPR) such as *Bacillus subtilis* on the plant roots.

In the present study, we systematically examined cyanogenesis in different strains of *P. aeruginosa* and *P. fluorescens* for its phytotoxicity by following a bioassay driven approach using the model plant *Arabidopsis thaliana*. We demonstrated that both indirect and direct exposure of pseudomonads and cyanide causes inhibition of *Arabidopsis* primary root growth. Further, we show that mechanistically, cyanide completely suppresses the auxin signalling mechanism, causing inhibition of the primary root growth. In addition, we show that pseudomonad cyanogenesis also causes suppression of beneficial rhizospheric processes such as colonization and biofilm formation by the biocontrol bacteria *Bacillus subtilis* in the *A. thaliana-B. subtilis* model system.

## Materials and Methods

### Plant material and chemicals

*Arabidopsis thaliana* wild type cultivar Columbia (Col-0) seeds were procured from Lehle Seeds (Round Rock, TX, USA). The *Arabidopsis* line stably expressing the DR5: GUS reporter fusion was obtained from Dr. Thomas Guilfoyle, University of Missouri, Columbia, MO 65211. KCN was obtained from Fluka, Germany.

### Culture conditions

Seeds were washed in double distilled water three times and surface sterilized using 50% commercial bleach (sodium hypochlorite) for 3–5 min followed by 3–4 washes in sterile distilled water. The seeds were cultured on Murashige and Skoog's [35] (MS) solid medium with 3% sucrose and allowed to germinate for 3–4 days by incubating at 23±2°C under 16 hr light and 8 hr dark. The plates were illuminated with cool fluorescent light with an intensity of 24 µmol m^−2^ s^−1^.

### Microbial strains and culture conditions

The *P. aeruginosa* strains PAO1, PA14 (obtained from Dr. Frederick Ausubel, Department of Molecular Biology, Massachusetts General Hospital, Boston, MA) were grown on Luria broth agar (LB) plates with 20 µg ml^−1^ rifampicin. The *P. fluorescens* strains CHAO, CHAO77 (cyanide synthase (Δ*hcnABC*) mutant), *P. aeruginosa* cyanide synthase mutant (Δ*hcnB*) PAO6344 and quorum sensing mutant PAO210 derived from PAO1 parent strain (obtained from Dr. Dieter Haas, Department of Fundamental Microbiology, Universite de Lusanne, Switzerland) were grown on LB plates. The *B. subtilis* wild type strain FB17 (obtained from Dr. Ray Fall, University of Colorado, Boulder) and strains with biofilm operon-lacZ transcription fusions, Marburg *thrC::yqxM-lacZ* (NRS1531) and Marburg *thrC::epsA-lacZ* (NRS1663) (obtained from Dr. Nicola R. Stanley-Wall, Division of Applied and Environmental Biology, School of Life Sciences, University of Dundee, Dundee, Scotland ) were grown on LB plates supplemented with 0.5 µg ml^−1^ erythromycin. A single colony from a freshly streaked plate (from glycerol stocks maintained at −80°C) with or without antibiotic selection of each of the cultures was used to grow overnight cultures from which approximately 0.02–0.05 OD_600_ culture was prepared and used in all the experiments.

### Vertical plate assay to study the direct effect of pseudomonad strains on the growth of *A. thaliana* Col-0 roots

MS solid medium (with 3% sucrose) plates containing a strip of LB solid medium were prepared by cutting out a strip of MS medium from pre-made MS plates and replacing it with LB solid medium. The LB strip was spotted with 10 µl of 0.02–0.05 OD_600_ cultures of different strains (of *P. aeruginosa*, *P. fluorescens*) and grown overnight in LB liquid medium with or without antibiotic selection (prepared by inoculating a single colony from a freshly streaked plate). The plates were incubated overnight at 37°C and the next day the plates were cultured with 3–4 day old *A. thaliana* Col-0 plants by laying them approximately 1.5 cm above the bacterial colony and the plates were incubated vertically at 23±2°C. The plant growth in terms of primary root length was measured on 5^th^ day and the data was expressed as total primary root length in centimeters.

### Compartment plate assay to study the indirect effect of pseudomonad strains on the growth of *A. thaliana* Col-0 roots

The indirect effect of pseudomonad strains on *A. thaliana* Col-0 roots was studied by using compartment plates. Of the two opposite compartments, one was filled with MS solid medium with 3% sucrose and the other was filled with LB solid medium. The compartment with LB medium was inoculated by spotting 10 µl of 0.02–0.05 OD_600_ cultures of different strains (of *P. aeruginosa*, *P. fluorescens*) grown in LB liquid medium with or without antibiotic selection. The plates were incubated overnight at 37°C and the next day the opposite compartment with MS solid medium was cultured with 3–4 day old *A. thaliana* Col-0 plants. Plants were added by laying them vertically, opposite to the compartment with the bacterial colony. The plates were made airtight by sealing with parafilm and incubated vertically at 23±2°C. The plant growth in terms of primary root length was measured on 5^th^ day and the data was expressed as total primary root length in centimeters.

### Kinetics of cyanide production in different pseudomonad strains

The kinetics of cyanide production at different time points (0–36 hr) was studied by growing the pseudomonad strains in 10 ml of LB broth. The culture was initiated by adding 2 µl culture (0.02 OD_600_) of each strain, prepared from overnight grown cultures. The cultures were incubated in a shaking incubator maintained at 37°C and set at 220 rpm. To estimate the cyanide content, a set of three culture flasks were removed at each time point and centrifuged to pellet out the cells. The cyanide content in the supernatant was then estimated by using ISM-146CN; C (micro combination ion cyanide electrode) procured from Lazar research Laboratories, Inc, Los Angeles, CA-90038, USA, by following a previously reported protocol [36]. The micro combination ion cyanide electrode is a combination electrode not requiring a separate reference electrode and can measure in volumes as low as <10 µl. Measurements were made by connecting electrode to a Mettler-Toledo MP220 pH meter set to read on mV. The cyanide content was calculated using a standard curve developed using standard KCN and data was presented as µM of cyanide ions produced. All the treatments had three replicates and the experiment was completed on two independent occasions.

### Cyanide sensitivity assay on the growth of *A. thaliana* Col-0 roots

The effect of cyanide on *Arabidopsis thaliana* Col-0 root growth was studied by using both the direct plate assay and the compartment plate assay described above except that the bacterial culture was replaced with KCN (0–700 µM). The filter disc soaked in a required amount of KCN from the stock was directly placed at a distance of 1.5 cm below the growing root tip of *A. thaliana* Col-0 plants. The indirect effect of cyanide was studied using compartment plates by soaking the sterile filter discs with KCN (0–700 µM KCN). A control filter disc soaked with sterile water used in both the assays. Further, 3–4 day old *Arabidopsis* seedlings were placed vertically on MS solid medium in the opposite compartment. The plates were incubated for five days, plant growth in terms of primary root length was measured on 5^th^ day and the data was expressed as total primary root length in centimeters.

### Histochemical assay for β-glucouronidase (GUS) activity

Three-day old seedlings of *A. thaliana* Col-0 transgenic line stably expressing a DR5::GUS fusion were used to study the indirect effect of pseudomonad metabolites and cyanide on auxin responsive gene expression in *Arabidopsis*. The experimental set up was similar to that of the compartment plate assay conducted to study the effect of indirect exposure of the pseudomonad strains and cyanide except Col-0-DR5::GUS seedlings were used instead of wild type Col-0. Other treatment sets such as culturing DR5::GUS seedlings on MS solid medium containing IAA (1 mg L^−1^), IAA+cyanide, transferring back 48 hr cyanide exposed plants back to IAA medium, were also included to study the effect of exogenously supplied IAA on DR5 expression. At the end of 5 days of incubation, the roots were stained for DR5 driven GUS expression using a GUS staining kit procured from Sigma, USA. The staining was performed according to the manufacturer's instructions. The stained roots were imaged using a stereo- microscope (Zeiss Axioskop-2). The resulting images were captured and processed using the Axiovision software.

### Effect of Pseudomonad Cyanide on *B. subtilis* FB17 Colonization and Biofilm Formation on *A. thaliana* Col-0 roots

Three-day-old seedlings of *A. thaliana* Col-0 germinated on MS solid medium with 3% sucrose were transferred to 4 wells of the six well plates containing 4 ml MS liquid medium with 1% sucrose. The other two wells were filled with LB solid medium. The plants were grown for an additional 9 days; on the 9^th^ day the wells with LB solid medium were inoculated with a 10 µl of 0.02 OD_600_ cultures each of the different strains used in the study, each strain to separate plates. The cultures were allowed to grow for one more day and on the 10^th^ day the wells with the plants were supplemented with 5 µl of 0.02 OD_600_ overnight grown cultures of *B. subtilis* FB17. Another set of plates were also set up where the two wells without the plants were used to generate cyanide gas by adding 20 µl each of 0.1 mM KCN and 0.1 mM HCl to the sterile filter discs placed in the wells. The plates were made airtight by sealing with parafilm, incubated on a rotary shaker set at 90 rpm, 24 hour post-inoculated roots were fixed in 4% para-formaldehyde and used for visualization and imaging for biofilm formation using confocal scanning laser microscope (CSLM).

### β-Galactosidase assay

To study the effect of pseudomonad cyanogenesis on the transcription profile of either the *yqxM* and *epsA* promoters under biofilm formation conditions, *B. subtilis* strain Marburg carrying either the *epsA-lacZ* fusion (NRS1663) or *yqxM-lacZ* fusion (NRS1531) were utilized. The *B. subtilis* strains were grown to mid-late exponential phase in biofilm medium [37] from a freshly streaked LB plate and subsequently diluted to an OD_600_ of 0.02 in fresh biofilm medium. 100 µl of the diluted culture was transferred to a flat-bottomed 96-well microtitre plate. Two rows on either side of the *B. subtilis* culture were supplemented with 100 µl 0.2 OD_600_ culture of different pseudomonad strains, each in a separate microtitre plates. A separate set of plates was also prepared with generated HCN instead of pseudomonad cultures in the two rows on either side of the *B. subtilis* culture by adding 30 µl each of 0.1 mM KCN and 0.1 mM HCl. The control plates had 100 µl of LB liquid medium in the wells of two rows on either side of the row containing *B. subtilis*. The microtitre plates were made airtight by sealing with parafilm and were incubated at 37°C in an incubator shaker. During incubation, the samples were collected at 3hr time intervals from 3–12 hrs and the OD_600_ recorded at each time point was used as an indication of cell growth. The β-galactosidase units produced per minute were estimated using the following protocol. Briefly, about 300 µl of the culture drawn at each time point were centrifuged at 14000 rpm at 4°C for 20 min. The pellet was then re-suspended in 0.5 ml Z buffer (0.06 M Na_2_HPO_4_.7H_2_O, 0.04 M NaH_2_PO4.H_2_O, 0.01 M KCl, 0.001 M MgSO_4_.7H_2_O), pH adjusted to 7.0 (the buffer was supplemented with 2.9 µl ml^−1^ (∼0.05M) of β-mercapto-ethanol prior to use) and also a drop of toluene was added. The re-suspended pellet and buffer solution was then vortexed for 5 min to lyse the cells. To the lysate, 0.2 ml of ONPG (O-nitrophenyl- β-D-galactopyranoside) (4 mg ml^−1^ in Z-buffer) was added. The reaction was stopped by adding 0.5 ml 1M NaCO_3_ and the time length of the reaction was recorded. The supernatant was collected by spinning down the cell debris at 14000 rpm for 5 min and the OD_420_ was recorded. The specific activity of β-galactosidase in terms of Miller units produced per minute was calculated by using the formula; specific activity (Miller units) = 1000×[OD_420_/(t×v×OD_600_) where, t = time in minutes; v = volume of the cells assayed. The β-galactosidase activity values were plotted against time. A separate graph showing the growth of the culture at different time intervals was also plotted as OD_600_ against time. The experiment was repeated on at least 3 independent occasions and a representative plot is shown.

### Microscopy

In order to view adherent *B. subtilis* FB17 cells and biofilm on the root surface by confocal scanning laser microscopy (CSLM), the roots were stained with SYTO®13 (Invitrogen, Molecular Probes, Eugene, OR). Images were captured with a 10× objective on a Zeiss LSM 510 NLO attached to an Axiovert 200M with an automated stage microscope equipped with Zeiss LSM 510 software. Each experiment was repeated twice with three replicates each.

### Statistical analysis

All data were averaged from two separate experiments and further analyzed for variance using Microsoft Excel, followed by a Student's *t* test. The data means were considered significantly different at the probability of P<0.05 according to Fisher's least significant difference (LSD) test.

## Results

### Pseudomonads inhibit primary root growth of *Arabidopsis thaliana* Col-0 seedlings

**Figure 1:**
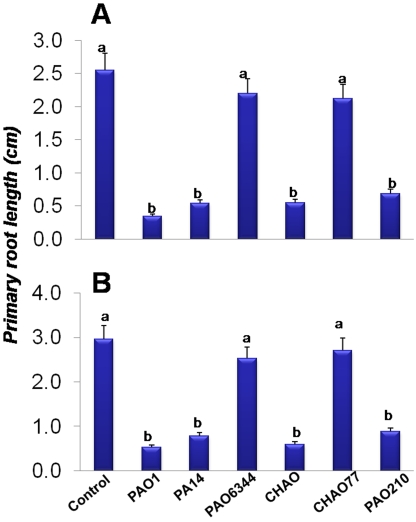
Direct and indirect effect of pseudomonad strains on the growth of *A. thaliana* Col-0 roots (A&B). Severe reduction of primary root length (A&B) followed by the death of the seedlings was observed 5 days post-inoculation in the case of PAO1, PA14 and CHAO cultured at a distance of 1.5 cm from the primary root tip. The data also shows less reduction in primary root length in the case of PAO6344, CHAO77, when compared to PA01, PA14 and CHAO, Different letters indicate a statistically significant difference between treatments (Fisher's LSD, *P*<0.05). Data is the mean±SD of 12 replicates and the experiment was repeated on two independent occasions.

The direct effect of pseudomonads on primary root growth was studied by inoculating 3–4 day old *A. thaliana* (Col-0) seedlings with different strains of bacteria at a distance of 1.5 cm from the primary root tip. The results presented in Figure 1A show that five days after inoculation, *P. aeruginosa* (strains PAO1&PA14) and *P. fluorescens* (wild type strain CHAO) caused significantly shorter primary root lengths in *A. thaliana* compared to control treatments (Fig. 1A&B) (P<0.05; n = 12). A less drastic (but not significant) reduction in the primary root length was observed with the cyanide synthase (Δ*hcnB*) mutant (PAO6344) of *P. aeruginosa* (Figure 1A&B) and CHAO77 (Δ*hcnABC*) of *P. fluorescens*. Apart from the wild type strains and cyanide mutants, we also examined a quorum sensing (QS) mutant PAO210 (Δ*rhlI*) of *P. aeruginosa* to evaluate if another known virulence pathway, such as QS played a role in the observed root phenotype. The data showed significant inhibition of primary root growth by the mutant PAO210 compared to the cyanide mutant indicating that the inhibitory effect is more of a result of the cyanide synthase mutation. These results also suggest that an external factor produced by pseudomonad strains is involved in the inhibition of *A. thaliana* Col-0 seedlings. However, the relatively less inhibition of root length by the cyanide mutant strains of *P. aeruginosa* (PAO6344) and *P. fluorescens* (CHAO77) indicated a possible role for cyanide in the inhibition of root growth.

### Indirect effect of Pseudomonad cyanogenesis causes the suppression of primary root growth of *A. thaliana* Col-0 seedlings

Most pseudomonad strains produce cyanide (CN^−^), which is subsequently converted to the diffusible HCN in aqueous media [24], [38]. To account for the effect of possible HCN emission from pseudomonad strains on *A. thaliana* Col-0 root growth, we conducted an indirect assay using compartment plates (see materials and methods for details). The data presented in Figure 1B shows that the *P. aeruginosa* strains PAO1, PA14, PAO210 and *P. fluorescens* strain CHAO caused a significantly higher suppression of primary root growth of *A. thaliana* Col-0 plants (P<0.05; n = 12) when compared to the untreated control plants even when the plants are spatially separated. Significantly less inhibition of primary root length was also observed with cyanide mutants PAO6344 and CHAO77 (P<0.05; n = 12). These results together with the previous direct assay results (Figures 1A) show that the pseudomonad exometabolites, specifically cyanide, inhibited the primary root growth of *A. thaliana* Col-0 plants.

### Pseudomonads produce cyanide (CN^−^) in cultures

**Figure 2:**
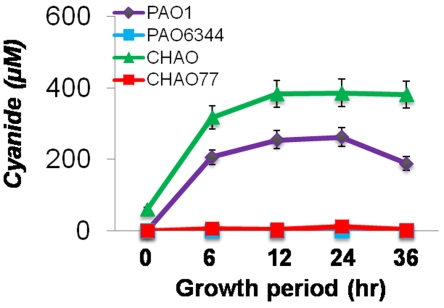
Kinetics of cyanide synthesis and accumulation in the different pseudomonad strains. The data shows highest cyanide accumulation in CHAO and PAO1 with an increased synthesis during late log phase and early stationary phase. The data is the mean±SD of 3 replicates and the experiment was repeated on two independent occasions.

Based on our earlier direct and indirect compartment plate bioassay results, we speculated that the primary root growth inhibition of *A. thaliana* Col-0 by different pseudomonad strains was due to cyanide production by these bacterial strains. Therefore, to check whether these strains produce cyanide in cultures, we estimated the total cyanide produced in the cultures of different strains of *P. aeruginosa* and *P. fluorescens.* The cyanide in the culture filtrate as CN^−^ ion was estimated using a specific cyanide ion electrode at different time intervals from 0 to 36 hours during the growth period. The results presented in Figure 2 show a significantly higher amount of cyanide production in the culture of *P. fluorescens* strain CHAO followed by *P. aeruginosa* strains PAO1 and PA14. However, significantly less cyanide production was observed in the cyanide mutants PAO6344, CHAO77 compared to the respective parental (PAO1 and CHAO) strains (Figure 2). The production of CN^−^ directly correlated with the root inhibition data described in Figure 1A&B. In most of the strains tested, the trend for cyanide accumulation in the cultures was highest in the early exponential phase followed by early stationary phase and accumulation declined in the late stationary phase.

### Cyanide inhibits primary root growth of *A. thaliana* Col-0 seedlings

**Figure 3:**
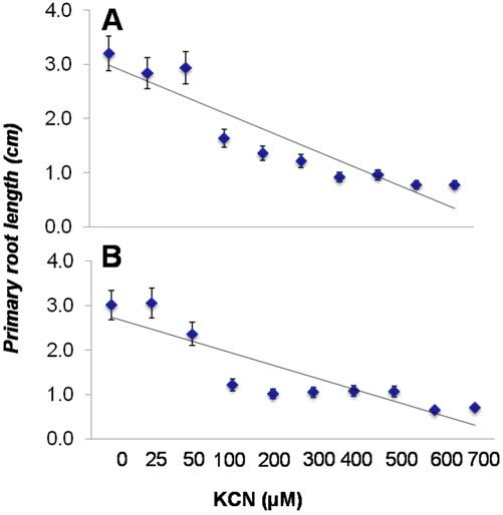
Direct and indirect effect of different doses (0–700 µM) cyanide on the growth of *A. thaliana* Col-0 seedling. The data shows the linear regression plots (A&B) shows the predicted (line) and experimental (dots) values of primary root length at different concentrations of KCN(B; *r* ^2^ = 0.8581) and HCN (D; *r* ^2^ = 0.779). Data is the mean±SD of 12 replicates and the experiment was repeated on two independent occasions.

Based on our earlier results, which showed a significantly higher cyanide ion accumulation in the cultures of *P. aeruginosa* PAO1/PA14 and *P. fluorescens* CHAO, we hypothesized that if the cyanide produced by the pseudomonad strains is responsible for primary root growth inhibition, exogenously supplied cyanide should also inhibit the *A. thaliana* Col-0 root growth. Consistent with our hypothesis, the plants exposed with both direct KCN and indirect (HCN) showed significant (P<0.05; n = 12) inhibition of the primary root growth of *A. thaliana* Col-0 seedlings (Figure 3A&B). The plot shows the linearity of the relationship between the predicted (line) and experimental (dots) values of primary root length and cyanide concentration (Figures 3A&B). Thus, all these results together conclusively established that the inhibition of *A. thaliana* Col-0 primary root growth by different strains of pseudomonads especially *P. aeruginosa* PAO1, PA14 and *P. fluorescens* CHAO is due to the production and release of cyanide. This result was also confirmed by the reduced inhibition of primary root growth by cyanide mutants of *P. aeruginosa* PAO6344 and *P. fluorescens* CHAO77 (Figures 1A&B).

### Pseudomonad cyanogenesis and cyanide down-regulate the expression of the auxin responsive promoter DR5

**Figure 4:**
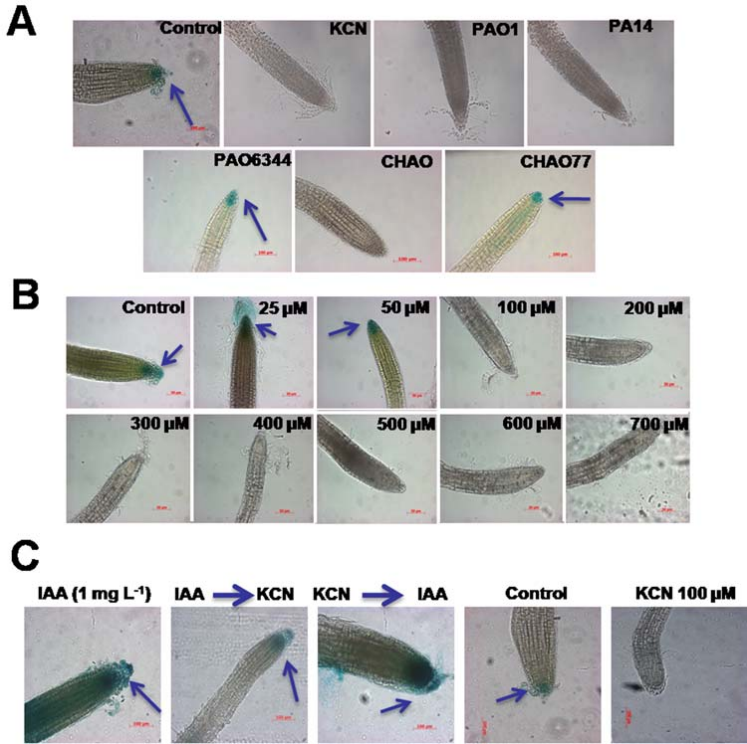
Suppression of DR5::GUS expression in *A. thaliana* by the indirect exposure of the pseudomonad strains (A) and cyanide (B) and the effect of exogenous IAA on cyanide mediated down regulation of DR5::GUS expression (C). The images show complete suppression of DR5::GUS expression in the Col-0 DR5::GUS transgenic seedling roots when indirectly exposed to *P. aeruginosa* strains PAO1 and PA14, CHAO and cyanide. The images were representative of ten independent plants imaged (A) (bar = 100 µm). The figure also shows the effect of different doses (0–700 µM) of cyanide on DR5::GUS expression. The arrows in the panel show the localized DR5 expression.

Auxin is an important plant hormone which controls primary root growth by regulating cell proliferation and enlargement [32]. Since all of our results with pseudomonad strains and cyanide showed severe inhibition of primary root growth in *A. thaliana* Col-0, we further speculated that this may affect the auxin biosynthesis/perception at the root tip. To examine this interesting speculation, we conducted compartment plate assays using an *A. thaliana* Col-0 transgenic line stably expressing a GUS reporter gene fusion to the auxin responsive promoter DR5 (DR5::GUS). The results presented in Figure 4A show a complete suppression of the DR5 expression when co-inoculated with *P. aeruginosa* strains PAO1/PA14, *P. fluorescens* strain CHAO and cyanide (KCN) treatment. All of the other bacterial treatments such as cyanide mutants PAO6344 and CHAO77, showed stable expression of DR5::GUS similar to the control untreated plants. The dose response experiments performed using HCN (0–700 µM KCN+0.1 M HCl) showed complete suppression of DR5::GUS expression at ≥100 µM (Figure 4B). Further, the DR5::GUS expression was not affected when exogenous IAA (1 mg l^−1^) was included in the assay plates and the DR5::GUS expression was restored in the plants pre-treated with cyanide and subsequently exposed to IAA (Figure 4C). These results suggest that the cyanide produced by different strains of *P. aeruginosa* cause the inhibition of primary root growth of *A. thaliana* Col-0 through a complete suppression of the auxin biosynthesis/perception at the root tip.

### Indirect exposure of the pseudomonad strains and cyanide suppress *Bacillus subtilis* colonization on *A. thaliana* Col-0 roots

**Figure 5:**
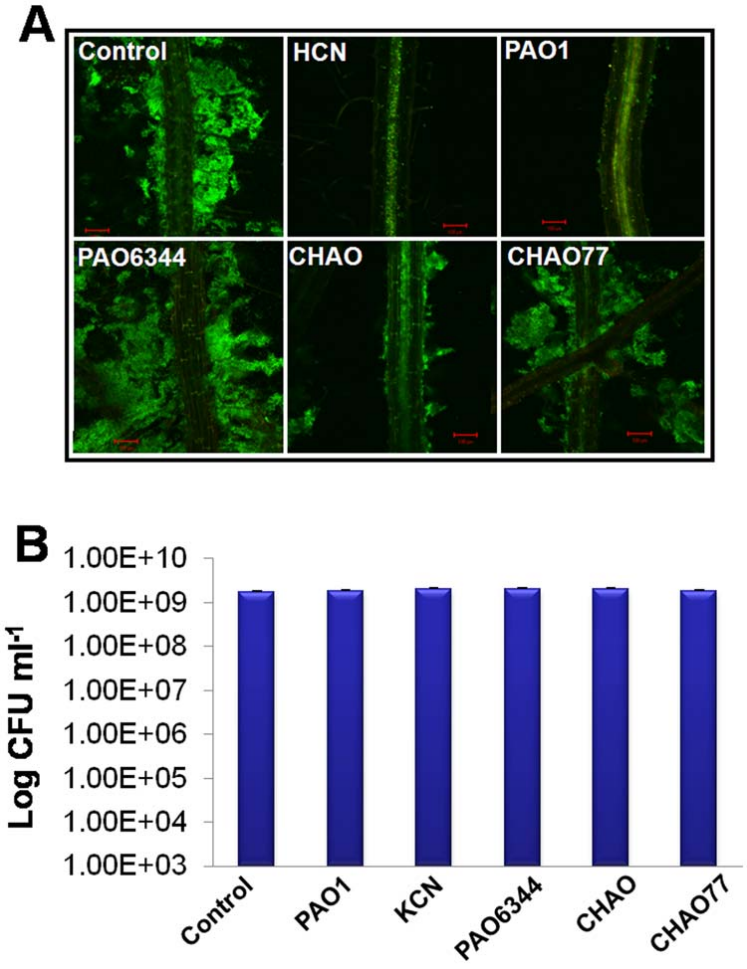
Suppression of *B. subtilis* biofilm formation on *A. thaliana* Col-0 roots by indirect exposure of the pseudomonad strains and cyanide (A) and the effect of indirect exposure of the pseudomonad strains and cyanide on single cell growth of *B. subtilis* (B). The images show complete suppression of *B. subtilis* biofilm formation when exposed to the indirect exposure of the strains PAO1, PA14, CHAO and HCN when compared to control plants not exposed to either bacterial culture or HCN and Δ*hcnB* mutants PAO6344 and CHAO77. The data also shows extensive colonization and biofilm formation by *B. subtilis* with indirect bacterial exposure from the *hcnB* mutants PAO6344 and CHAO77. The images were representative of the roots from six independent plants imaged.

Since the pseudomonads are common soil bacteria, we suspected that apart from their effect on plant roots, they might also influence other rhizospheric processes such as beneficial bacteria-root associations. Therefore, we studied the possible effects of pseudomonad cyanogenesis on *B. subtilis* colonization and biofilm formation on *Arabidopsis*. *B. subtilis*, a biocontrol PGPR (plant growth-promoting rhizobacteria), forms biofilms on *A. thaliana* roots and protects the plant from pathogenic infections [37], [39]. The experiment was conducted *in vitro*; in six well plates using 10-day-old *A. thaliana* seedlings grown in MS liquid medium with 1% sucrose (see materials and methods). The results presented in Figure 5 show a complete suppression of *B. subtilis* FB17 biofilm formation on the roots subjected to indirect exposure with *P. aeruginosa* strains PAO1/PA14, *P. fluorescens* strain CHAO and HCN (KCN+HCL) (Figure 5A). The control which did not receive any bacterial culture, and the treatments, with bacterial cultures of cyanide impaired mutants PAO6344, and CHAO77 formed extensive biofilms. Although indirect exposure of the pseudomonad strains and cyanide caused complete suppression of *B. subtilis* biofilm formation on *Arabidopsis* roots, they did not however, affect the single cell growth of *B. subtilis* in the *Arabidopsis* culture medium as indicated by FB17 colony forming units (CFU) data (Figure 5B). These results clearly indicated that apart from the inhibition of plant primary root growth, pseudomonad cyanogenesis also affects other rhizospheric processes such as biofilm formation by a beneficial biocontrol PGPR such as *B. subtilis.*

### Pseudomonad cyanogenesis and cyanide down regulate *Bacillus subtilis* biofilm operons

**Figure 6:**
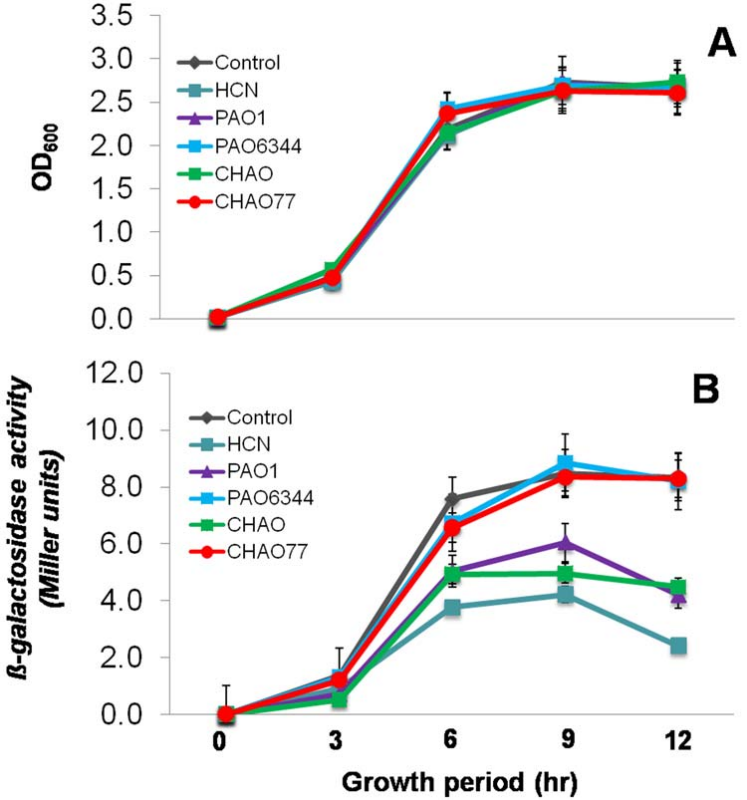
Effect of indirect exposure of the pseudomonad strains and cyanide on the transcription of the *epsA* operon in *B. subtilis.* Strain *Marburg thrC::epsA-lacZ* (NRS1663) was grown in biofilm medium under biofilm formation conditions at 37°C with or without exposure to pseudomonad strains and HCN. Growth (A) and β-galactosidase activity (B) were measured at regular intervals and plotted as a function of time. Data is the mean±SD of 12 replicates and the experiment was repeated on two independent occasions. These experiments were repeated on at least 3 independent occasions and a representative plot is shown.

**Figure 7:**
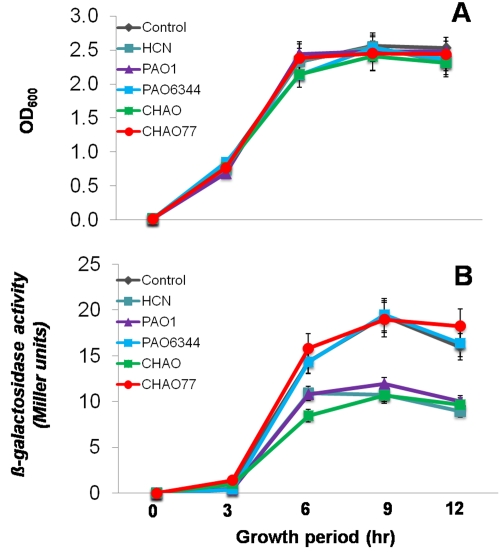
Effect of indirect exposure of the pseudomonad strains and cyanide on the transcription of the *yqxM* operon in *B. subtilis.* Strain *Marburg thrC*::*yqxM*-*lacZ* (NRS1531) was grown in biofilm medium under biofilm formation conditions at 37°C with or without exposure to pseudomonad strains and HCN. Growth (A) and β-galactosidase activity (B) were measured at regular intervals and plotted as a function of time. These experiments were repeated on at least 3 independent occasions and a representative plot is shown. Data is the mean±SD of 12 replicates and the experiment was repeated on two independent occasions.

While pseudomonads and cyanide suppressed the ability of *B. subtilis* to form biofilms, it did not suppress the single cell growth. Therefore, we hypothesized that pseudomonads and cyanide may inhibit biofilm formation through limiting the induction of one (or both) of two key loci required for biofilm formation, the *epsA-O* and *yqxM-sipW-tasA* operons [40]–[45]. To test this hypothesis, we used the transcriptional fusions of the promoter regions for both the *epsA* and *yqxM* operons to β-*galactosidase* and monitored the expression profile in the presence and absence of PAO1, CHAO, PAO6344, CHAO77 and cyanide. Treatment with PAO1, cyanide and CHAO suppressed the induction of *epsA* by more than 40% (Figure 6B) and *yqxM* by about 50% (Figure 7B) when compared to the untreated control and cyanide mutants. The suppression of transcription induction in each case occurred from the late log phase and continued through the stationary phase (see Figure 6A&7A for growth), suggesting that there may be a receptor that is actively produced in the late log-phase to late stationary phase in *B. subtilis* that could perceive the presence of cyanide. These data clearly showed the specificity of cyanide and cyanide producing pseudomonads in down regulation of *B. subtilis* biofilm operons.

## Discussion

Among the fluorescent pseudomonads, cyanogenesis in the soil bacteria *P. fluorescens* has been researched extensively on its role as a biocontrol agent [46]. However, the studies on the role of cyanogenesis in the virulence of *P. aeruginosa* are very scarce. Along similar lines, it has been reported that cyanide production in *P. aeruginosa* mediate paralytic killing of *C. elegans* in a fast killing assay [1]. To understand the role of cyanogenesis in a plant infection model system, we followed a systematic bioassay driven approach. As a first step towards this objective, we tested the effect of different pseudomonad strains (both *P. fluorescens* and *P. aeruginosa*) on the growth of *A. thaliana* Col-0 roots. The direct bacterial plate assay revealed that a diffusible factor produced by the pseudomonad strains resulted in acute primary root growth inhibition. However, a reduced growth inhibition was observed with the cyanogenic mutants PAO6344 and CHAO77, giving an indication for the involvement of cyanide in the growth inhibitory effect. Our observation was partially supported by the fact that cyanide is reported as a strong virulence factor of *P. aeruginosa* [1]. Similarly, the compartment plate assays demonstrated that indirect exposure of the strains exhibited a similar growth inhibitory phenotype as observed previously with the direct plate assays. In addition, exogenously supplied cyanide in the form of KCN and HCN caused similar root inhibitory effects. The cyanide in pseudomonads is produced by the oxidative decarboxylation of glycine by a membrane bound three-subunit enzyme encoded by *hcnABC* [20]. The reduced growth inhibitory effect observed with the indirect exposure of the two cyanide synthase mutant strains, one each from *P. aeruginosa* and *P. fluorescens*, indicated the direct role of cyanogenesis. However, the primary root growth inhibitory effect observed with the direct plate assay may be due to the interference of other virulence factors in the pseudomonads, in addition to cyanide. This observation was well supported with the quantitative data on cyanide production recorded in different pseudomonad cultures in the present study. The observed indirect effects in compartment plate assays are very important due to the fact that the microbe-synthesized cyanide is readily converted into the HCN form, diffuses into the air and exerts its effect on the flora and fauna in the soil [20]. It is reported that HCN is not required for growth, energy storage, or primary metabolism but may provide some ecological advantage to the organism [47]. The highest level of cyanide produced was observed in the late exponential and early stationary phase in both *P. aeruginosa* PAO1 and *P. fluorescens*, therefore, the present study was in accordance with earlier reports [20]. However, the slightly reduced recovery of roots from the inhibition effects in the case of cyanide mutants may be a result of the production of other virulence factors in addition to cyanide [15]–[17]. Whereas the effect in the case of PAO210 could be due to significantly higher hcnA expression, resulting in higher cyanide production as previously reported for mutation in *rhl* operon [48].

A mechanistic insight into the severe rhizotoxicity of cyanide on *A. thaliana* roots was envisaged by utilization of an artificial auxin responsive transgene (DR5). DR5, an artificial AuxRE containing the TGTCTC element, has increased auxin responsiveness to auxin [49]. The GUS reporter gene fused to a minimal cauliflower mosaic virus 35S promoter, and the DR5 has been used widely as a marker to monitor endogenous IAA distribution because the resulting GUS activity coincides with endogenous IAA distribution [50]–[51]. To gain insight into the mechanisms of cyanide-induced rhizotoxicity, we studied the involvement of the DR5 in cyanide-induced auxin expression. Transgenic *A. thaliana* seedlings containing the DR5-GUS reporter gene were exposed in compartment plate assays to different pseudomonad strains and HCN. Interestingly, the DR5::GUS seedlings treated with pseudomonad strains and HCN revealed a complete suppression of DR5::GUS expression at the root tip level. However, the restoration of DR5::GUS expression when supplemented with exogenous IAA further confirmed the involvement of cyanide mediated impairment of auxin biosynthesis and/or perception. A significant reduction in the seedling root growth of lettuce, barnyard grass and green foxtail was observed when treated with pseudomonad released metabolites, which was further correlated to HCN production [22]. However, there is no report on the effect of cyanide on either auxin gene expression or signaling in plant roots. Interestingly, it has been observed that the phytotoxic effect of the auxin herbicide quinclorac on shoot growth was mediated through the accumulation of phytotoxic levels of cyanide [52]. Further, this accumulation was caused through the induction of ACC synthase (1-aminocyclopropane-1-carboxylic acid) activity in the tissues, which produce cyanide as a co-product of ethylene [52], [53].

Plant respiration rates are usually amended in response to both biotic and abiotic stress [54]. Cyanide has been reported to play an important role in modulating the respiratory electron transport in aerobic respiration in all plants, many fungi and some protozoa [54]. Conversely, decrease in respiration rates, because of abiotic stress like cyanide exposure results in cell death [54]. This mortality effect is due to the inhibition of cytochrome *c* oxidase (COX) which prevents electron transport from COX to O_2_, resulting in impedance of proton motive force that drives ATP synthesis [55]–[57]. Additionally, it is known that inhibition of ATP synthesis affects auxin biosynthesis in terms of its transport and localization in plants [58].

It is reported that auxin can stimulate cell elongation in plant roots, which is usually accompanied by promotion of respiration [59], [60]. However, it is uncertain if respiration is promoted when auxin induces cell elongation. Our studies showed the cyanide mediated impairment of auxin signaling. It could be argued that our observation relates to an indirect effect of the impeded respiration post-cyanide treatment on auxin biosynthesis. Interestingly, a report on other respiration inhibitors (monoiodoacetate, malonate and 2,4-dinitrophenol) shows elimination of the auxin effect on respiration and cell division [61]. Based on previous studies and our findings, it can be concluded that promotion of respiration is a common auxin effect in plants [61] and impeding auxin biosynthesis could lead to respiration inhibition and cell death. In the future, it would be interesting to study the relationship between cyanide-mediated suppression of auxin induced gene expression and the reported mechanism of cyanide mediated inhibition of mitochondrial respiration [62].

Apart from its effect on root growth, the cyanide produced by pseudomonad strains in the soil has been reported to cause inhibitory effect on pathogenic fungi and bacteria [63], [64]. Except for this ecological role, there are no reports available which demonstrate the effects of pseudomonad cyanogenesis on rhizosphere microorganisms. Our study shows the effect of cyanide on the *B. subtilis* colonization and biofilm formation on the roots of *A. thaliana*. Our recent reports suggest that *B. subtilis* FB17 forms a complete and mature biofilm on *A. thaliana* roots [39], [65]. The biofilm formation ability of *B. subtilis* is related to its biocontrol activity against root borne pathogens [39]. Our results indicated a suppression of *B. subtilis* FB17 colonization by the indirect exposure of the pseudomonad strains and cyanide without affecting the *B. subtilis* single cell growth. Further, the suppression of the *B. subtilis* biofilm operons *epsA* and *yqxM* by indirect exposure of pseudomonads and cyanide supported this observation. These results suggest that the effect is brought about through the down regulation of key biofilm operons in *B. subtilis*. This intriguing result signifies the plausible role of cyanogenesis in regulating various rhizospheric interactions. However, another possibility for the observed non-lethality of released cyanide on the single cell growth of *B. subtilis* maybe due to partial degradation of the released cyanide, keeping bacterial exposure below the lethal dose. A cyanide dihydratase capable of degrading the cyanide has been reported in *Bacillus pumilis* [66].

In summary, the present report established that pseudomonad cyanogenesis affects *A. thaliana* Col-0 primary root growth by inhibiting auxin synthesis/perception. Additionally, this study established that cyanogenesis also affected one of the multitrophic rhizospheric processes; in particular, *B. subtilis* biofilm formation on *Arabidopsis* roots *in vitro*. Therefore, this study highlights the multifaceted attributes of a bacterial virulence factor, capable of harming its host through more than one mechanism and also implicates cyanogenesis as a multi-host virulence factor. Since these studies are conducted, *in vitro,* future studies would benefit from testing other host and testing the infection processes in host's microcosm. It would also be interesting to study the effects of cyanogenesis in other root- rhizospheric microbial interactions and its effect on the root exudation profile as a marker for perturbation in overall plant defense.

## References

[1] Gallagher LA, Manoil C (2001). *Pseudomonas aeruginosa* PAO1 Kills *Caenorhabditis elegans* by cyanide poisoning.. Journal of Bacteriology.

[2] Rodgers PB, Knowles CJ (1978). Cyanide production and degradation during growth of *Chromobacterium violaceum.*. Journal of General Microbiology.

[3] Castric PA, Vennesland B, Conn EE, Knowles CJ, Westley J, Wissing F (1981). The metabolism of hydrogen cyanide by bacteria.. Cyanide in biology.

[4] Vennesland B, Pistorius EK, Gewitz HS, Vennesland B, Conn EE, Knowles CJ, Westley J, Wissing F (1981). HCN production by microalgae.. Cyanide in biology.

[5] Knowles CJ, Bunch AW (1986). Microbial cyanide metabolism.. Advances in Microbial Physiology.

[6] Antoun H, Beauchamp CJ, Goussard N, Chabot R, Lalande R (1998). Potential of *Rhizobium* and *Bradyrhizobium species* as plant growth promoting rhizobacteria on non-legumes: effect on radishes (*Raphanus sativus* L.).. Plant and Soil.

[7] Castric PA (1977). Glycine metabolism by *Pseudomonas aeruginosa*: hydrogen cyanide biosynthesis.. Journal of Bacteriology.

[8] Rennert T, Mansfeldt T (2002). Sorption of iron-cyanide complexes on goethite in the presence of sulfate and desorption with phosphate and chloride.. J Environ Qual.

[9] Govan JR, Deretic V (1996). Microbial pathogenesis in cystic fibrosis: mucoid *Pseudomonas aeruginosa* and *Burkholderia cepacia.*. Microbiology Reviews.

[10] Jander G, Rahme LG, Ausubel FM (2000). Positive correlation between virulence of *Pseudomonas aeruginosa* mutants in mice and insects.. Journal of Bacteriology.

[11] Mahajan-Miklos S, Tan MW, Rahme LG, Ausubel FM (1999). Molecular mechanisms of bacterial virulence elucidated using a *Pseudomonas aeruginosa-Caenorhabditis elegans* pathogenesis model.. Cell.

[12] Rahme LG, Stevens EJ, Wolfort SF, Shao J, Tompkins RG (1995). Common virulence factors for bacterial pathogenicity in plants and animals.. Science.

[13] Silo-Suh L, Suh S-J, Sokol PA, Ohman DE (2002). A simple alfalfa seedling infection model for *Pseudomonas aeruginosa* strains associated with cystic fibrosis shows AlgT (sigma-22) and RhlR contribute to pathogenesis.. Proceedings of the National Academy of Sciences, USA.

[14] Wood WB (1988). The nematode *Caenorhabditis elegans*.

[15] Lyczak JB, Cannon CL, Pier GB (2000). Establishment of *Pseudomonas aeruginosa* infection: lessons from a versatile opportunist.. Microbes and Infection.

[16] Terada LS, Johansen KA, Nowbar S, Vasil AI, Vasil ML (1999). *Pseudomonas aeruginosa* hemolytic phospholipase C suppresses neutrophil respiratory burst activity.. Infection and Immunity.

[17] Budzikiewicz H (1993). Secondary metabolites from fluorescent pseudomonads.. FEMS Microbiology Reviews.

[18] Britigan BE, Railsback MA, Cox CD (1999). The *Pseudomonas aeruginosa* secretory product pyocyanin inactivates protease inhibitor: implications for the pathogenesis of cystic fibrosis lung disease.. Infection and Immunity.

[19] Olvera C, Goldberg JB, Sanchez R, Soberon-Chavez G (1999). The *Pseudomonas aeruginosa algC* gene product participates in rhamnolipid biosynthesis.. FEMS Microbiology Letters.

[20] Blumer C, Haas D (2000). Mechanism, regulation, and ecological role of bacterial cyanide biosynthesis.. Archives of Microbiology.

[21] Walker TS, Bais HP, De'ziel E, Schweizer HP, Rahme LG (2004). *Pseudomonas aeruginosa*-Plant root interactions. Pathogenicity, biofilm formation, and root exudation.. Plant Physiology.

[22] Bano N, Musarrat J (2003). Characterization of a new *Pseudomonas aeruginosa* strain NJ-15 as a potential biocontrol agent.. Curr Microbiol.

[23] De Vleesschauwer D, Cornelis P, Höfte M (2006). Redox-active pyocyanin secreted by *Pseudomonas aeruginosa* 7NSK2 triggers systemic resistance to *Magnaporthe grisea* but enhances Rhizoctonia solani susceptibility in rice.. Mol Plant Microbe Interact.

[24] Brinker AM, Seigler DS (1989). Methods for the detection and quantitative determination of cyanide in plant materials.. Phytochemical Bulletin.

[25] Tattersal DB, Bak S, Jones PR, Olsen CE, Nielsen JK (2001). Resistance to an herbivore through engineered glucoside synthesis.. Science.

[26] Hayden KJ, Parker IM (2002). Plasticity in cyanogenesis of *Trifolium repens* L. inducibility, fitness costs and variable expression.. Evolutionary Ecology Research.

[27] Ballhorn DJ, Lieberei R, Ganzhorn JU (2005). Plant cyanogenesis of *Phaseolus lunatus* and its relevance for herbivore-plant interaction: The importance of quantitative data.. Journal of Chemical Ecology.

[28] Begonia MFT, Kremer RJ (1994). Chemotaxis of deleterious rhizobacteria to velvet leaf (*Abutilon theophrasti* Medik.) seeds and seedlings.. FEMS Microbiology Ecology.

[29] Kremer RJ, Kennedy AC (1996). Rhizobacteria as biocontrol agents of weeds.. Weed Technology.

[30] Kremer RJ, Souissi T (2001). Cyanide production by rhizobacteria and potential for suppression of weed seedling growth.. Current Microbiology.

[31] Alström S, Burns RG (1989). Cyanide production by rhizobacteria as a possible mechanism of plant growth inhibition.. Biology and Fertility of Soils.

[32] Fukaki H, Okushima Y, Tasaka M (2007). Auxin-mediated lateral root formation in higher plants.. International Review of Cytology.

[33] Taylor LP, Grotewold E (2005). Flavonoids as developmental regulators.. Current Opinion in Plant Biology.

[34] Fu X, Harberd NP (2003). Auxin promotes *Arabidopsis* root growth by modulating gibberellin response.. Nature.

[35] Murashige T, Skoog F (1962). A revised medium for rapid growth and bioassay with tissue culture.. Physiologia Plantarum.

[36] Zlosnik JEA, Williams HD (2004). Methods for assaying cyanide in bacterial culture supernatant.. Letters in Applied Microbiology.

[37] Rudrappa T, Quinn WJ, Stanley-Wall NR, Bais HP (2007). A degradation product of the salicylic acid pathway triggers oxidative stress resulting in down-regulation of *Bacillus subtilis* biofilm formation on *Arabidopsis thaliana* roots.. Planta.

[38] Zeller SL, Brandl H, Schmid B (2007). Host plant selectivity of rhizobacteria in a crop/weed model system.. PLoS One.

[39] Bais HP, Fall R, Vivanco JM (2004). Biocontrol of *Bacillus subtilis* against infection of *Arabidopsis* roots by *Pseudomonas syringae* is facilitated by biofilm formation and surfactin production.. Plant Physiology.

[40] Hamon MA, Stanley NR, Britton RA, Grossman AD, Lazazzera BA (2004). Identification of *AbrB*-regulated genes involved in biofilm formation by *Bacillus subtilis*.. Molecular Microbiology.

[41] Branda SS, Gonza'lez-Pastor JE, Ben-Yehuda S, Losick R, Kolter R (2001). Fruiting body formation by *Bacillus subtilis*.. Proceedings of the National Academy of Sciences, USA.

[42] Branda SS, Gonzalez-Pastor JE, Dervyn E, Ehrlich SD, Losick R (2004). Genes involved in formation of structured multicellular communities by *Bacillus subtilis*.. Journal of Bacteriology.

[43] Branda SS, Chu F, Kearns DB, Losick R, Kolter R (2006). A major protein component of the *Bacillus subtilis* biofilm matrix.. Molecular Microbiology.

[44] Chu F, Kearns DB, Branda SS, Kolter R, Losick R (2006). Targets of the master regulator of biofilm formation in *Bacillus subtilis*.. Molecular Microbiology.

[45] Kearns DB, Chu F, Branda SS, Kolter R, Losick R (2005). A master regulator for biofilm formation by *Bacillus subtilis*.. Molecular Microbiology.

[46] Askeland RA, Morrison SM (1983). Cyanide production by *Pseudomonas fluorescens* and *Pseudomonas aeruginosa*.. Applied and Environmental Microbiology.

[47] Vining LC (1990). Functions of secondary metabolites.. Annual Review of Microbiology.

[48] Pessi G, Haas D (2000). Transcriptional control of the hydrogen cyanide biosynthetic genes *hcnABC* by the anerobic regulator ANR and the quorum sensing regulators *LasR* and *RhlR*.. Journal of Bacteriology.

[49] Ulmasov T, Hagen G, Guilfoyle TJ (1997). ARF1, a transcription factor that binds to auxin response elements.. Science.

[50] Sabatini S, Beis D, Wolkenfelt H, Murfett J, Guilfoyle T (1999). An auxin-dependent distal organizer of pattern and polarity in the *Arabidopsis* root.. Cell.

[51] Casimiro I, Marchant A, Bhalerao RP, Beeckman T, Dhooge S (2001). Auxin transport promotes *Arabidopsis* lateral root initiation.. Plant Cell.

[52] Grossmann K (2004). Mediation of herbicide effects by hormone interactions.. Journal of Plant Growth Regulation.

[53] Grossmann K, Schmülling T (1995). The effects of the herbicide quinclorac on shoot growth in tomato is alleviated by inhibitors of ethylene biosynthesis and by the presence of an antisense construct to the 1-aminocyclopropane-1-carboxylic acid (ACC) synthase gene in transgenic plants.. Plant Growth Regulation.

[54] Siedow JN, Umbach AL (2000). The mitochondrial cyanide-resistant oxidase: structural conservation amid regulatory diversity.. Biochim Biophys Acta.

[55] Buchnan BB, Balmer Y (2005). Redox regulation: a broadening horizon.. Annual Review of Plant Biology.

[56] Umbach AL, Ng VS, Siedow JN (2006). Regulation of plant alternative oxidase activity: a tale of two cysteines.. Biochimica Biophysica Acta.

[57] Raghavendra AS, Padmasree K (2003). Beneficial interactions of mitochondrial metabolism with photosynthetic carbon assimilation.. Trends in Plant Science.

[58] Caldogno FR, Cerana R, Pugliarello MC (1978). Effects of anaerobiosis on auxin-induced growth and ion transport.. Cellular and Molecular Life Sciences (CMLS).

[59] Audus LJ, Ruhland W (1960). Effect of growth regulating substances on respiration.. Encyclopedia of plant physiology XII, pt 2.

[60] Bonner J, Bandurski RS (1952). Studies on physiology, pharmacology and biochemistry of auxin.. Annual Review of Plant Physiology.

[61] Leonova LA, Gamburg KZ, Vojnikov VK, Varakina NN (1985). Promotion of respiration by auxin in the induction of cell division in suspension cultures.. Journal Plant Growth Regulation.

[62] Albury MS, Dudley P, Watts FZ, Moore AL (1996). Targeting the plant alternative oxidase protein to *schizosaccharomyces pombe* mitochondria confers cyanide-insensitive respiration.. Journal of Biological Chemistry.

[63] Voisard C, Bull CT, Keel C, Laville J, Maurhofer M, O'Gara F, Dowling DN, Boesten B (1994). Biocontrol of root diseases by *Pseudomonas fluorescens* CHA0: Current concepts and experimental approaches.. Molecular ecology of rhizosphere microorganisms.

[64] Laville J, Blumer C, Von Schroetter C, Gaia V, Défago G (1998). Characterization of the *hcnABC* gene cluster encoding hydrogen cyanide synthase and anaerobic regulation by ANR in the strictly aerobic biocontrol agent *Pseudomonas fluorescens* CHA0.. Journal of Bacteriology.

[65] Rudrappa T, Bais HP (2007). *Arabidopsis thaliana* root surface chemistry regulates in planta biofilm formation of *Bacillus subtilis.*. Plant Signaling & Behavior.

[66] Jandhyala D, Berman M, Meyers PR, Sewell BT, Willson RC (2003). CynD, the cyanide dihydratase from *Bacillus pumilus*: Gene cloning and structural studies.. Applied and Environmental Microbiology.

